# Food protein-induced allergic proctocolitis in infants: Literature review and proposal of a management protocol

**DOI:** 10.1016/j.waojou.2020.100471

**Published:** 2020-10-01

**Authors:** Maurizio Mennini, Alessandro Giovanni Fiocchi, Arianna Cafarotti, Marilisa Montesano, Angela Mauro, Maria Pia Villa, Giovanni Di Nardo

**Affiliations:** a Multifactorial and Systemic Diseases Research Area, Predictive and Preventive Medicine Research Unit, Division of Allergy Bambino Gesù Children's Hospital IRCCS, Rome, Italy; b Chair of Pediatrics, NESMOS Department, Faculty of Medicine and Psychology, Sapienza University of Rome, Sant’Andrea University Hospital, Rome, Italy; c Department of Paediatrics, Santobono-Pausilipon Children's Hospital, Naples, Italy

**Keywords:** Allergic colitis, Eosinophils, Rectal bleeding

## Abstract

Food protein-induced allergic proctocolitis (FPIAP) is a condition characterized by inflammatory changes in the distal colon in response to one or more foreign food proteins because of immune-mediated reactions.

FPIAP prevalence estimates range widely from 0.16% in healthy children and 64% in patients with blood in stools.

In clinical practice, FPIAP is diagnosed when patients respond positively to the elimination of a suspected triggering food allergen. Nevertheless, significant proportions of infants get misdiagnosed with IgE mediated allergy and undergo unnecessary dietary changes.

Diagnosis is based on clinical symptoms, a good response to an allergen-free diet and the recurrence of symptoms during the “allergy challenge test”. Sometimes clinical features may be non-specific and the etiology of rectal bleeding in childhood may be heterogeneous. Therefore, it is crucial to exclude a variety of other possible causes of rectal bleeding in the pediatric age group, including infection, anal fissure, intestinal intussusception and, in infants, necrotizing enterocolitis and very early onset inflammatory bowel disease. The diagnostic workup includes in those cases invasive procedures such as sigmoidoscopy and colonoscopy with biopsies.

The high prevalence of FPIAP contrasts with the lack of known information about the pathogenesis of this condition. For this reason and due to the absence of a review of the evidence, a literature review appears necessary to clarify some aspects of allergic colitis.

The aim of the review is to fill this gap and to lay the foundations for a subsequent evidence-based approach to the condition.

## Introduction

The high prevalence of food protein-induced allergic proctocolitis (FPIAP) in healthy subjects and patients with rectal bleeding contrasts with a lack of known information about the pathogenesis of this condition. For this reason, and due to the absence of a review of the evidence, a literature review appears necessary to clarify some aspects of allergic colitis.

Due to the heterogeneity of the methods within the resources, it was not possible to conduct a literature review with a systematic approach.

The aim of the review is to fill this gap and to lay the foundations for a subsequent evidence-based approach to the condition.

### Definition

Allergic colitis, also known as eosinophilic proctocolitis or food protein-induced allergic proctocolitis (FPIAP), is characterized by inflammatory changes in the distal colon in response to one or more different food proteins because of immune-mediated reactions. The underlying mechanism is not known, though IgE is not implicated.^1^

The symptoms typically begin in the first months of life and most cases, resolve by late infancy. Infants with FPIAP usually present with red blood and mucus mixed with the stool, with or without diarrhea. Some of them are fussy and irritable; most affected infants are generally healthy appearing. Patients frequently do not have weight loss or impairment of the general condition or anomalies at abdominal physical examination.^2^

FPIAP is a clinical entity and the diagnosis is often made presumptively. There is no accurate diagnostic exam available. Various non-specific markers can be used, as well as the disappearance of clinical symptoms when suppressing the culprit food, with clinical relapse when the food is reintroduced. It is occasionally necessary to perform invasive exams such as endoscopy with biopsy.^3^ Endoscopy generally shows focal or diffuse colitis, with edema and erosions.

Rectal bleeding is not a rare problem in otherwise healthy breast-fed infants.^4^ The differential diagnosis includes anal fissures, intussusception, infectious colitis, necrotizing enterocolitis, and very early onset inflammatory bowel disease.^5^

Cow's milk and sometimes soy is the most common trigger for FPIAP, though it is commonly characterized by multiple hypersensitivities to food. The infant may be exposed to the cow's milk protein through breast milk or infant formula. The elimination diet is the essential method of treating allergic enteritis. Gross rectal bleeding should resolve within 72–96 h, but the total resolution of symptoms takes 1–2 weeks from the beginning of the diet.^6^

## Pathogenesis of allergic proctocolitis

The exact immunologic mechanisms responsible for this condition have not yet been clarified. It is usually described as a non-IgE–associated disease.^7^

Food antigen sensitization seems to play a key role in the development of allergic colitis. FPIAP may follow the absence of immunological food tolerance.^8^ Several studies have shown that several immune system cells take part in oral tolerance induction.^8^ A research on children with multiple food allergies demonstrated that the main immunological anomaly in the small bowel is failure to establish normal levels of transforming growth factor β (TGF-β)- producing regulatory cells.^9^ It has been hypothesized that failure to produce Th3 cells, because of insufficient innate immune response by commensal intestinal microbes, could contribute to an impaired development of oral tolerance.^10^

In allergic colitis of older children, the main abnormality was the reduced production of TGF-β. A former research on toddlers with multiple food allergies showed by flow cytometry, immunohistochemistry, and *in situ* hybridization that the expression of TGF-β significantly decreased in the duodenal mucosa with either immediate or delayed adverse food reactions.^12^ Ozen et al support that a reduced expression of TGF-β might be implicated in the sensitization of children to dietary proteins.^11^^,^^12^

TNF-α is another pivotal cytokine associated with chronic inflammatory diseases. It has been demonstrated to alter the tight junctions between epithelial cells. This property of TNF-α has been suggested in the pathogenesis of allergic colitis in children, possibly by altering the epithelial intestinal barrier capacity. In line with these evidence, a research examined concentrations of TNF-α in 3 patient groups: control patients; IgE-mediated cow's milk allergy patients; and gastrointestinal food allergy patients (including allergic colitis). The study measured TNF-α concentrations in peripheral blood mononuclear cells of each patient group and found that patients with gastrointestinal food allergies had significantly greater TNF-α concentrations than those seen in other groups. Concentrations of the Th1 cytokine IFN-γ and the Th17 cytokine IL-17 did not show statistically significant differences between any 2 groups. Th2 cytokines (IL-3, IL-5 and IL-13) were significantly produced *in vitro* by milk protein-stimulated PBMCs from patients with gastrointestinal food allergies.^13^

Other studies have suggested a possible role of T cells. The interplay between CD28 on T cells and its 2 ligands B7-1 (CD80) and B7-2 (CD86) on antigen presenting cells is considered the main co-stimulatory pathway for generating a T cell response. In animal models of allergic airway inflammation and hypersensitivity to peanut, studies have shown that CD86 ligand stimulation of CD28 T cells leads to the development of an IgE response, whereas a CD80 ligand stimulation of CD28 T cells leads to the induction of low-dose tolerance to peanut.^14^ However, further studies are needed to better understand the relationship between allergens and cell-mediated response in allergic proctocolitis.

Histologic examinations in allergic proctocolitis often reveal focal aggregates of eosinophils in the large intestinal epithelium, lamina propria, crypt epithelium, and muscularis mucosa. Furthermore, multinucleated giant cells have occasionally been identified in the submucosa. Eosinophils are a prominent subtype of leukocytes differentiated from multipotent hematopoietic stem cells from myeloid lineage myeloblasts.^15^ Eosinophils are multifunctional leukocytes involved in innate and adaptive immune responses.^16^, ^17^, ^18^ One study showed that eosinophils home into the digestive tract in the prenatal period.^20^ Eosinophils set up inflammatory and adaptive responses because of their interactions with antigen presenting cells and T cells. They have also shown an ability to synthesize numerous cytokines and mediators. Augmented level of eosinophils in tissue or blood with remarkable degranulation is reported in many inflammatory diseases (eosinophilic dermatitis, asthma, gastroesophageal reflux disease, celiac disease, inflammatory bowel disease, and parasitic infections). In normal conditions, eosinophils are found in every segment of the gastrointestinal tract from the stomach to the colon in the lamina propria except the esophagus, Peyer's patches, and intra-epithelial locations.^19^, ^20^, ^21^ Eosinophils reply to a variety of chemoattractant: eotaxin-1, -2, -3, RANTES, MIP-1α, MCP-2, -3, -4, and lipid mediators like PAF, LTB4, LTC4.^22^

The specificity of eotaxin for eosinophils is the consequence of the exclusive signaling of eotaxin-1, eotaxin-2, and eotaxin-3 through its receptor, CCR3, which is expressed predominantly on human and mouse eosinophils. Eosinophil-selective chemokine, eotaxin-2, and eotaxin-3 have 30% homology to eotaxin-1 and were recently detected.^23^

FPIAP can often be a demonstration of other primary diseases.

Further research is required to understand the role of several costimulatory molecules and subsets of dendritic cells in the induction of oral tolerance versus allergic sensitization.

## Epidemiology

FPIAP prevalence estimates range widely from 0.16% in healthy children and 64% in patients with blood in stools.^24^^,^^25^

In a large prospective population-based study, the prevalence of rectal bleeding attributed to cow's milk protein is 1.6 per 1000 infants.^25^ A United States-based study performed a prospective cohort study of infants with rectal bleeding, demonstrating that 64% are diagnosed with FPIAP, based on biopsy findings from flexible sigmoidoscopy.^25^ A Finnish study confirms FPIAP in only 18% of infants presenting with rectal bleeding, when milk elimination and challenge was used to make the diagnosis.^26^

Allergic colitis is believed to be a common cause of rectal bleeding in healthy infants, but the true prevalence has not been established because of the absence of a specific diagnostic test. Most cases are empirically diagnosed and treated.^3^

FPIAP typical age of onset is from days to 6 months, it usually occurs in young infants within the first 2 months of life. It is a transitory disease, which in most cases disappears around the first year of life. Older children and adults with allergic colitis to cow milk (CM), egg, and wheat have been rarely described.^25^, ^26^, ^27^

It was reported that cow's milk proteins (65%) are the most common triggers responsible for the development of the symptoms of allergic enteritis, but also egg, corn, soy, and/or wheat can be implicated (in 19%, 6%, and 3%, respectively). Moreover about 5% of infants have an identified multiple food allergy.^28^ Kaya et al showed that in their cohort, cow's milk was the offending allergen in all 60 patients diagnosed with FPIAP.^29^

The infant may be exposed to the cow's milk protein through breast milk or infant formula. More than 50% of cases of FPIAP reported in literature are exclusively breast-fed infants, and in most cases a gradual and complete resolution of the disease can be observed after 72–96 h of maternal avoidance of offending proteins. The breast-fed infants who do not respond to maternal dietary restriction improve after weaning to an extensively hydrolyzed or amino acid-based formula.^25^^,^^26^^,^^28^^,^^29^ In less than 10% of the cases, extensively hydrolyzed formulas may induce FPIAP symptoms.^27^ In formula fed infants the diet is typically changed to a protein hydrolysate formula (PHF) and subsequently to an elemental l-amino acid (LAA) formula if bleeding does not resolve.^25^

The prognosis of FPIAP is generally good, indeed up to 20% of breastfed infants have spontaneous resolution, and nearly all infants become tolerant to the culprit food by 1 to 3 years of age.^24^^,^^30^

## Non-invasive exams

In clinical practice, FPIAP is diagnosed when patients respond positively to the elimination of a suspected triggering food allergen. Nevertheless, a significant proportion of infants get misdiagnosed with IgE mediated allergy and undergo unnecessary dietary changes.

Below we describe non-invasive exams, which have been considered to confirm diagnosis of allergic proctocolitis in suspected patients.

### Skin prick tets, patch test, serum IgE, and IgG4

Little research has evaluated the role of skin prick tests, patch tests, and serum IgE evaluation.^31^^,^^32^ Although IgG and IgG4 measurements are trialed in adult studies, there is no evidence for clinical validity of any biomarkers for the diagnosis of non–IgE-mediated food allergy in childhood. Consensus documents have pointed out the need for taking an allergy-focused history and use this to perform the diagnosis.

Biomarkers have performed poorly across the spectrum of non–IgE-mediated allergies. The use of atopy patch testing has been proposed to work out "delayed sensitization";^33^ however, the latter test has produced contradictory evidence.^34^ The atopy patch test has also shown inconsistency in predicting when tolerance has been achieved in non-IgE CMA.^35^^,^^36^ Consequently, international guidelines do not recommend patch testing as a routine test for the diagnosis of non–IgE-mediated allergies.^37^

Equally, IgG and IgG4 testing have little settled clinical validity, and they are used only in research studies, alongside mucosal inflammatory markers and tests of gastrointestinal permeability^38^^,^^39^

A position paper of the European Academy of Allergy and Clinical Immunology (EAACI) on Non IgE mediated allergy stated that IgE testing may be taken into consideration in breastfed infants with symptoms associated with IgE-mediated allergies, comorbid presentations such as atopic dermatitis, and after a long period of avoidance before food reintroduction.^40^

The meta-analysis by Lozinsky et al showed that 43.8% of 263 infants with allergic proctocolitis had blood eosinophilia and eosinophilic infiltration in colonic mucosa in 89.3%. In addition to eosinophilia, the study found a decreased ratio of interferon-γ/IL-4, an increase in Th2 lymphocytes, and a decrease in regulatory T lymphocytes.^2^ However, none of these findings are specific to FPIAP, but rather demonstrate an atopic state or other allergic diseases.

### Fecal occult blood test (FOBT)

Diagnostic validity of fecal occult blood test (FOBT) was assessed in infants with rectal bleeding secondary to allergic proctocolitis compared to healthy infants. The results of this study showed that, although FOBT has adequate sensitivity (84%; negative predictive value 83%), it has inadequate specificity (66%; positive predictive value 68%) since more than one-third of healthy infants had positive FOBT.^41^

Several studies have shown a high prevalence of positive fecal occult blood test (FOBT) in healthy infants and non-allergic diseases. It was observed that in a group of 180 infants who were hospitalized due to non-gastrointestinal diseases, 34% of healthy infants showed positivity to FOBT, thus reaffirming the given the high number of false positives. The low specificity of the FOBT shows the inapplicability of using this test as a non-invasive marker of allergic proctocolitis.^39^

### Stool smear

Another diagnostic test for FPIAP is a stool smear for eosinophilic granules. However, the diagnostic validity has also been poor. The stool smear cannot differentiate between allergic proctocolitis and food protein-induced enterocolitis because both diseases can have the presence of eosinophilic granules on stool smear.

In a retrospective analysis of 64 infants (Mean±SD: 1.68 ± 1.01 months) in whom a diagnosis of diarrhea disorder had been made after an initial investigation. All infants received fiberoptic sigmoidoscopy and mucosal biopsy. Colon mucosa pathologic findings were found: 40 (62.5%) had eosinophilic colitis and 19 (29.7%) had nonspecific colitis. Eosinophils in the stool smear was significantly higher (p = 0.04) in FPIAP compared to nonspecific colitis (9/17).^7^

### Fecal calprotectin

Calprotectin is a calcium and zinc-binding protein that accounts for 60% of the cytosol proteins in neutrophils and crucial for the clearance of infection.^42^

It was demonstrated that fecal calprotectin (FC) is elevated in infants with hematochezia. Positive FC was significantly higher (p < 0.0001) in infants suspected of having cow milk allergic colitis compared to healthy infants. Furthermore, they described a significant decrease in positive FC tests in infants after 4 weeks of dietary antigen elimination. Importantly, levels of positive FC remained significantly higher in treated patients with cow milk allergic colitis compared to in age- and diet-matched healthy infants. However, a significant decrease in fecal calprotectin was also observed in the age- and diet-matched healthy infant group.^43^

FC concentration reflects the inflammation of the intestinal mucosa permeability. For example, it has been demonstrated that FC levels in stools are higher in preterm infants with gastrointestinal bleeding. Blood transports not only erythrocytes but also neutrophils, regardless of whether there is evidence of inflammation or not. Therefore, it is common to find higher FC levels in the presence of bleeding, even if the blood is present in traces.^44^, ^45^, ^46^, ^47^, ^48^

It is described that FC levels have a non-parametric distribution and should be evaluated with appropriate statistical analysis. There is an age-dependent variation in FC.^49^, ^50^, ^51^ Roca M et al suggested 3 different age groups for evaluation of FC concentrations. The 95th percentile for the following age groups 0–12 months, >1–4 years and >4–12 years was 910.3 mg/kg, 285.9 mg/kg, and 54.4 mg/kg respectively. The authors concluded that healthy children have higher FC concentrations than healthy adults.^52^

Furthermore, it was showed how fecal calprotectin, should not be used routinely because there is no significant correlation between positivity of allergy tests, fecal calprotectin, endoscopic score, histological score, or eosinophil score in FPIAP.^53^

### Ultrasound and color Doppler ultrasound

In pediatrics, ultrasound (US) and color Doppler ultrasound (CDUS) have been increasingly used to evaluate intestinal inflammation. Intestinal inflammatory diseases cause thickening of the intestinal wall that can be revealed using US. A retrospective analysis of US reports of 13 infants diagnosed with allergic proctocolitis showed that 12 (92.3%) out of 13 infants had an abnormal US. The positive US findings suggesting colitis were increased vascularity and thickened bowel walls, particularly in the descending and sigmoid colon. Colonoscopy and histopathological investigations were performed in these 13 infants and the findings were compatible with allergic proctocolitis.^54^ All 13 patients were then placed on an exclusion diet, and 7 out of 13 infants had a repeated US after exclusion diet. The repeated US showed a grayscale and color Doppler sonographic change, which was suggestive of an improvement in vascularization and thickness of the bowel wall.

The physiopathology of allergic colitis in infants is generally linked with intestinal inflammation, and ultrasound may, therefore, detect these abnormalities. When associated with clinical parameters, ultrasound may be useful to suggest the diagnostic hypothesis.

In a study, the role of Doppler US was to assess vascular alterations in the intestinal wall of infants with abdominal pain and rectal bleeding. Doppler US characteristics help to confirm the presence of colitis, with clinical findings suggestive of FPIAP and ruling out other abdominal diseases, such as intestinal intussusception.

Nevertheless, US and CDUS findings are not specific, as they can also be found in infectious colitis. However, correlating these US findings with color Doppler, clinical data, and laboratory tests could enable to confirm diagnosis. The right colon is the best part to identify inflammation at US and CDUS. Inflammation predominantly at rectum and sigmoid could not be visualized.

The competence of the ultrasonographist is crucial to get valid results. The main limit of this method is reproducibility, as it is operator dependent. Cut points for the CDUS findings should be established in large sample of patients for an effective application of this technique.

Doppler US has been applied in the evaluation of abdominal pain and intestinal bleeding, and could be a tool for FPIAP. The presence of increased colonic wall thickness and vascularity at Doppler could be indicative of intestinal inflammation, adding a pivotal element in the correct clinical setting.

## Invasive exams

Diagnosis is based on clinical features, a good response to an allergen-free diet and the recurrence of symptoms during the “allergy challenge test”. Sometimes clinical features may be non-specific and the etiology of rectal bleeding in childhood may be heterogeneous. Therefore, it is very important to exclude a variety of other possible causes of rectal bleeding in the pediatric age group, including infection, anal fissure, intestinal intussusception and, in infants, necrotizing enterocolitis, and early onset inflammatory bowel disease (IBD).^5^

The diagnostic workup includes invasive procedures such as sigmoidoscopy and colonoscopy with biopsies.

Several studies have confirmed the diagnostic importance of these techniques in identifying allergic colitis.^55^

More recently, Dehghani SM et al studied 730 children with lower gastrointestinal bleeding subjected to colonoscopy and histopathology, and described many features of various intestinal diseases diagnosed in the study's population. This epidemiological study revealed that, due to heterogeneous differential diagnosis of rectal bleeding in children, the performance of sensitive and specific procedures in order to confirm the diagnosis, such as colonoscopy and histopathology, is recommended.^56^

On occasion, an endoscopy may show non-specific features of allergic colitis such as focal erythema, loss of vascular pattern, ulceration, or diffuse nodularity, or it may be normal. For this reason and because FPIAP is usually a patchy disease, multiple biopsies must be obtained for this diagnosis.^57^ In the literature, a very rare case is described in which allergic colitis showed features of granulomatous components. In particular, Dargent et al described a case of allergic colitis in which endoscopy showed nodular lymphoid hyperplasia, a high number of eosinophils that had infiltrated the lamina propria and the presence of a granulomatous infiltrate, histocytes, and multinucleate giant cells involving submucosa.^58^

To date, several studies have demonstrated the importance of eosinophil (Eo) infiltrating the intestinal mucosa and lamina propria.^59^^,^^60^

Finally, Hurrel et al aimed to provide practical data for the diagnosis of eosinophilic esophagitis and gastrointestinal disorders made by histopathological pictures. With regards to allergic proctocolitis in infancy, they firstly premise that, in healthy people, the density of Eo in the colon varies according to the different segment (ranging from 10 to 70 Eo/HPF in the cecum to 1 to 30 in the rectum) and then assume that, histologically in FPIAP, the architecture of mucosa is preserved and the eosinophilic infiltration is typically localized in the rectum. They also suggest that more than 60 Eo/10 HPFs in the lamina propria and eosinophilic infiltration in the epithelium or the muscularis mucosae are suggestive of eosinophilic proctocolitis.^61^

## Allergic colitis treatment

Current method of treating FPIAP is the elimination of presumed triggering antigens. Cow's milk proteins are most commonly involved, although multiple food allergens can be implicated.^62^ In a breastfeeding infant, it is important to support the beneficial role of breastfeeding, but cow's milk proteins should be eliminated from the maternal diet. Clinical bleeding typically clears within 1 to 2 weeks with complete elimination of the offending protein from the diet of the mother. Most cases resolve within 72–96 h. If the child is still symptomatic at least 2 weeks after the start of the diet it is firstly necessary check the mother's adherence to the diet and then to eliminate soy, followed by egg from the maternal diet.^63^

If more than one food protein is restricted from the diet of the breastfeeding mother, it will need supervision by a dietitian to ensure nutritional adequacy and to prevent excess weight loss in the mother. Occasional recurrence of bleeding is common in breastfed infants, probably because of inadvertent maternal intake of small amounts of the triggering protein. If the bleeding is infrequent, minor, and self-limited, it is reasonable to take no variation other than ongoing vigilance to maintain the current level of dietary restriction.

It was showed that patients with FPIAP often do not have weight loss or impairment of the general state or changes of palpation of the abdomen. An early diagnosis and a proper nutrition intervention will allow the infant to maintain the rate of growth and promote the complete disappearance of the symptoms.^2^

Although it is reported in about 12% of cases of FPIAP, offending foods could not be identified through maternal dietary variation, and breast feeding maintenance led to intermittent persistent bleeding.^28^ The optimal management for this group of infants has not been established and should be considered on a case-by-case basis. The options are: to continue breastfeeding despite ongoing symptoms or to switch from breastfeeding to a hydrolyzed or amino acid-based formula.

The first choice may be appropriate for mothers who find the dietary restrictions to be very burdensome, or those who were considering stopping breastfeeding for other reasons. Lucarelli et al^64^ enrolled 14 exclusively breast-fed infants with FPIAP, which did not resolve with an oligoantigenic maternal diet. Breastfeeding was discontinued and exclusive feeding with an amino acid-based formula (AAF) was started prior to trying an extensively hydrolyzed formula (eHF) as guidelines would recommend^62^ because of the potential allergenicity of eHF, due to residual immunologically active proteins.

The infants showed progressive clinical and endoscopic resolution. The initial non-responsivity may have been due to multiple food allergies, as shown by both the numerous sensitization detected by atopy patch test (APT) in 50% of patients and the APT positivity for breast milk of mothers on a hypoallergenic diet.

The second alternative is controversial but may be appropriate for infants with mild symptoms if the mother is committed to breastfeeding despite the need for dietary restrictions. The risks for the infant are not well defined but are probably low. Several studies^2^^,^^30^^,^^65^ reported that up to 20% of breastfed infants with AC have spontaneous resolution of bleeding without changes in the maternal diet and that the AP long-term prognosis is excellent. The risk of anaemia seems to be very low.

In 2018^66^ a new proposal for the management of the FPIAP depending on whether the duration of hematochezia was proposed. In case of hematochezia with duration less than or equal one month, they suggest waiting for the spontaneous resolution without elimination diet; in case of a period of more than one month, they suggest an elimination diet and, if hematochezia disappears, a challenge. If after the challenge hematochezia reappears, they suggest resuming the elimination diet for 3 months. In formula fed infants the diet is typically changed to a protein hydrolysate formula (PHF). In some infants bleeding does not resolve with this change and the diet is changed to an elemental l-amino acid (LAA) formula that is even more expensive.^25^ Changing to a soy-based formula is not generally recommended because a significant percentage of children who are sensitive to cow's milk are also sensitive to soy protein. This combined sensitivity probably occurs in at least 15% of infants [96]; earlier reports suggested the proportion might be as high as 40%.^24^^,^^62^^,^^64^

Very limited evidences suggest that probiotic supplement (e.g., Lactobacillus rhamnosus GG) may promote recovery or acquisition of tolerance.^67^

Arachidonic acid (AA), one of the polyunsaturated fats being supplemented in infant formulas, is the principal precursor of various inflammatory cytokines. Studies suggest that AA may accentuate allergic reactions by further fueling the inflammation. Teitelbaum JE illustrates the development of allergic colitis in an infant after supplementation with AA.^68^ Clearly, more studies are needed to determine if the addition of AA and docosahexaenoic acid carries risks that outweigh the benefits.^68^ While manufacturers strive to make a formula that resembles breast milk, one should consider that breast milk is inherently hypoallergenic and therefore greater concentrations of AA may be better tolerated than in a formula with foreign/antigenic proteins.

Resolution of FPIAP usually occurs during infancy.^63^ For infants who become asymptomatic after elimination of cow's milk or other suspected antigenic protein, the standard approach is a gradually reintroduction of the protein at 1 year of age, and this is usually successful. It is usually endorsed to reintroduce the offending food/s to the mother's or infant's diet after it has been eliminated for 6 months or at 12 months of age. In some circumstances, it may be possible to successfully early reintroduce the offending protein^26^. In 2012 it was described that after 3 months of the beginning of the diet, the large part of children tolerated the guilty food.^28^ In 2015, Nowak-Wegrzyn apprised that the reintroduction of the offending food within the first 6 months generally induces recurrence of bleeding. Nowak-Wegrzyn suggested that with negative skin prick test (SPT) and serum food-specific IgE antibody levels, food introduction could take place at home with gradual increase from 1 oz/day to full feedings over 2 week. Furthermore, up to 20% of breastfed infants have spontaneous resolution of hematochezia without mother's elimination diet.^30^ Indeed, the prognosis of FPIAP is excellent, nearly all infants become tolerant by one to 3 years of age and the majority achieves clinical tolerance by 1 year.^26^

Sigmoidoscopy and colonoscopy with multiple biopsies are a useful implement for further evaluation of patients. This test is usually reserved for patients with atypical symptoms to rule out other possible diagnosis or with severe rectal bleeding or anemia despite a trial of cow's milk elimination diet.^25^

### Outcome of allergic colitis

In previous studies, AC was identified as a risk factor for functional gastrointestinal disorders (FGIDs), supporting the existence of “postinflammatory” FGIDs.^69^, ^70^, ^71^ Allergic proctocolitis, a cause of rectal bleeding in exclusively breast-fed, constitutes an elegant human model of colitis, with similarities to early-life inflammation in animal studies.^25^^,^^64^^,^^72^^,^^73^ In an animal model of neonatal maternal separation, stress induces visceral hypersensitivity and increased pain perception via mast cell degranulation, nerve growth factor, and transient receptor ion channel one modulation, in the absence of overt - physiological mechanisms.^74^ Adult studies suggest that an abnormal mucosal milieu and neuro-immune interactions, caused by mast cell activation and nerve growth factor release, may be identified as crucial mechanisms in the pathophysiology of intestinal dysfunction resting to note that the kind of inflammation, as well as the period of life in which it occurs, are pivotal in defining its long-term effects.^75^ Two research groups have recently shown that with inflammatory bowel disease have an FGIDs prevalence like that of the general pediatric population.^76^, ^77^, ^78^, ^79^

It was demonstrated that an early-life event of allergic/inflammatory origin may be the trigger for the onset of persistent digestive symptoms, particularly IBS. Early in life, the intestine is characterized by an altered intestinal permeability, an immature immune system, and a sensitive stage of microbiotic development, with complex interactions between host and microbiota.^53^

In this crucial phase, an early disruption of gut homeostatic equilibrium, such as FPIAP, might predispose to susceptibility to the onset of FGIDs later in life.

### Proposal of a management protocol

The use of sigmoidoscopy and colonoscopy, associated with multiple biopsies, plays a crucial rule in the diagnosis of this disease and the differential diagnosis of rectal bleeding. Nevertheless, there are no specific recommendations on the timing of colonoscopy in children with suspected FPIAP.

The current method of treating FPIAP is the elimination of presumed triggering antigens. Nevertheless, there are no specific protocols for management of diets.

**Fig. 1:**
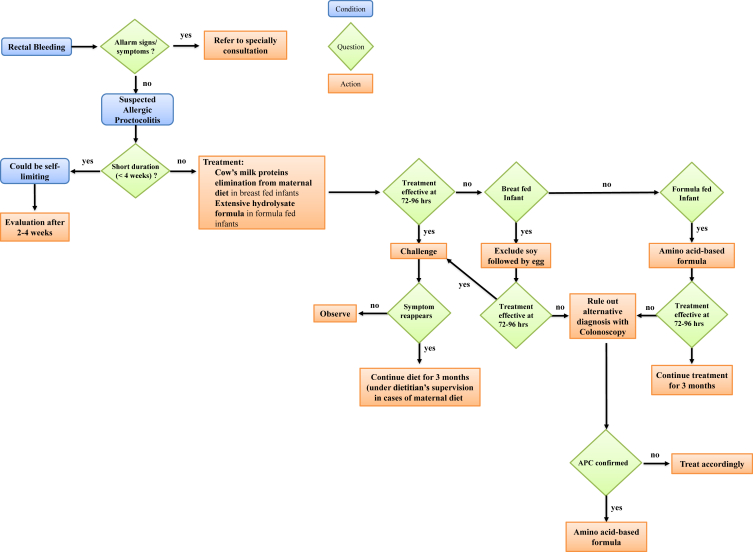
Protocol of diagnosis and management of FPIAP. Alarm signs and symptoms: severe perianal disease, abdominal distension, growth failure, anaemia, poor general conditions

We propose a possible protocol of diagnosis and management of FPIAP, following the main evidences (Fig. 1).

## Abbreviations section

Food protein-induced allergic proctocolitis: FPIAP
Transforming growth factor β: TGF-β
Cow milk: CM
Protein hydrolysate formula: PHF
l-amino acid: LAA
Fecal Occult Blood Test: FOBT
Fecal calprotectin: FC
Ultrasound: US
Color Doppler ultrasound: CDUS
Amino acid-based formula: AAF
Extensively hydrolyzed formula: eHF
Inflammatory Bowel Disease: IBD
Atopy patch test: APT
Arachidonic acid: AA
Skin prick test: SPT
Functional Gastrointestinal Disorders: FGIDs

## Acknowledgements

None.

## Funding

The authors did not receive any funding to conceptualize or realize this publication.

## Consent for publication

All the authors give the consent for publication in the journal.

## Ethics approval

Ethics approval was not required for this literature review.

## Author contributions

Maurizio Mennini and Giovanni Di Nardo conceived the review and research method of bibliographic sources. Maurizio Mennini, Arianna Cafarotti, Marilisa Montesano and Angela Mauro performed the research, the analysis and the selection of the sources.

Alessandro Giovanni Fiocchi and Maria Pia Villa performed the critical analysis of the sources and the final revision of the manuscript. Maurizio Mennini and Giovanni Di Nardo wrote the first draft of the manuscript.

All the authors accepted the final version of the manuscript.

## Availability of data and materials

The manuscript is a non-systematic review of the literature. The sources cited are then available and indicated in the list of references.

## Declaration of competing interest

None. I have read Elsevier's guidance on competing interests and have included a statement indicating that none of the authors have any competing interests.
